# Budd-Chiari Syndrome Transplant, Meso-Atrial Shunt or Combined Portocaval Shunt With Cavo-Atrial Shunt

**DOI:** 10.1155/1993/75272

**Published:** 1993

**Authors:** K. E. F. Hobbs

**Affiliations:** University Department of Surgery The Royal Free Hospital School of Medicine Pond Street London NW3 2QG USA


17. Kobe, E., Zipprich, P., Schentke, K-U. and Nilius, R. (1990) Prophylactic endoscopic sclerotherapy of esophageal varices — a prospective controlled randomized trial. *Endoscopy*, **22**, 245–248
18. The Veterans Affairs Cooperative Variceal Sclerotherapy Group (1991) Prophylactic sclerotherapy for esophageal varices in men with alcoholic liver disease: a randomized, single blind, multicenter clinical trial. *New Engl.J.Med.*, **324**, 1779–1784
19. Trigger, D.R., Smart, H.L., Hoskin, S.H.W. and Johnston, A.L. (1991) Prophylactic sclerotherapy for esophageal varices: long-term results of a single center trial. *Hepatology*, **13**, 117–123
20. de Franchis, R., North Italian Endoscopic Club (1991) Prophylactic sclerotherapy in high risk cirrhotics selected by endoscopic criteria — a multicenter randomized controlled trial. *Gastroenterology*, **101**, 1087–1093

### BUDD-CHIARI SYNDROME - TRANSPLANT, MESO-ATRIAL SHUNT OR COMBINED PORTOCAVAL SHUNT WITH CAVO-ATRIAL SHUNT

### ABSTRACT

*Orloff, M.J. and Daily, P.O. (1992) Treatment of Budd-Chiari syndrome due to inferior vena cava occlusion by combined portal and vena caval decompression. The American Journal of Surgery; 163: 137–143.*

**This study concerns Budd-Chiari syndrome (BCS) caused by occlusion of the subdiaphragmatic inferior vena cava (IVC). It describes the experimental and clinical evaluation of the treatment of this disorder by one-stage combined portal and vena caval decompression with a direct side-to-side portacaval shunt (PCS) and a caval-atrial shunt (CAS) graft. BCS was produced in rats by gradual occlusion of the suprahepatic IVC with an ameroid constrictor. When ascites and portal hypertension were established, 12 control rats survived a sham thoracolaparotomy, 16 rats survived a mesoatrial shunt, and 16 rats survived combined PCS and CAS graft. All control rats re-formed ascites and died within 2 months. Nine of 16 rats with mesoatrial shunt developed graft thrombosis, re-formed ascites, and died within 2 months. In contrast, only 2 of 16 rats that underwent combined PCS and CAS developed graft thrombosis, re-formed ascites, and died. Liver biopsies showed reversal of severe pathologic changes in rats with patent grafts. Clinical evaluation of combined PCS and CAS using a 20-mm ring-reinforced Gore-Tex graft has been undertaken in five patients with BCS and ascites, hepatosplenomegaly, intense hepatic congestion on biopsy, and angiography showing occlusion of both the IVC and hepatic veins. All five patients were alive and well 6 months to 7.5 years postoperatively with patent grafts, no ascites or need for diuretics, no encephalopathy, normal liver function, and reversal of liver pathology. It is concluded that combined PCS and CAS create a high-flow shunt that decompresses both the portal system and IVC, has a low incidence of graft thrombosis, has been consistently effective in relieving BCS caused by IVC occlusion, and appears to be superior to mesoatrial shunt.**

**PAPER DISCUSSION**

KEY WORDS: Budd-Chiari Syndrome, meso-atrial shunt, portocaval shunt, cavo-atrial shunt, liver transplantation

The combination of clinical features known collectively as the Budd-Chiari syndrome results from obstruction to the venous drainage of the liver. There are several causes for this but they all produce hepatic venous congestion, varying degrees of hepato-cellular dysfunction and a rise in portal pressure. The obstruction can be anywhere between the venules of the liver and the inferior vena cava. The natural history of the illness both in mode and speed of presentation and the spectrum of the resulting symptoms can vary from a severe acute condition and early death, to a chronic condition of cirrhosis, portal hypertension and bleeding oesophageal varices, in some cases many years later.

Since the syndrome has multiple aetiologies, it is not surprising that there is no single therapeutic approach. Unfortunately many clinicians do not seem to appreciate this and many apparently different approaches have been described with varying degrees of success since they are usually unrelated to the aetiology. These vary from conservative, "Let's hope it will recover spontaneously" to liver transplantation. Each proponent claims success for his approach. The Second International Conference on Budd-Chiari Syndrome debated this in Kyoto, Japan in the autumn of 1991 and concluded that the syndrome needs logical classification in order to propose rational treatment[1].

In general there are two different pathological processes which can lead to this syndrome: disseminated intra-hepatic veno-occlusive disease and hepatic vein and vena cava blockage. The first affects the venules in the liver usually secondary to exposure to some thrombogenic toxin such as plant alkaloids, cytotoxic drugs or some myeloproliferative disorder. The second either follows thrombotic or neoplastic processes which occlude the lumen or cause pressure on the vessels, or results fram an anatomical abnormality of the cava, the so called caval web. This may be congenital or develop in early infancy, and is seen mainly in Asian patients, being uncommon in the West.

Diagnosis, especially the acute form of the disease, must be established rapidly. The definitive tests include liver biopsy, hepatic angiography with venous pressure measurements or Doppler ultrasound of the cava and liver vessels. Histology will demonstrate the classical centrilobular congestion and the imaging and pressure measuring techniques will demonstrate obstruction to the hepatic venous flow.

Once the diagnosis of acute Budd-Chiari syndrome has been confirmed conservative management is ineffective and denying the patient interventional treatment is dangerous. The one caveat to this is the possibility of using thrombolytic therapy in acute veno-occlusive disease in an attempt to lyse the clot[2]. This certainly demands further study especially in relation to the possible use of recombinant tissue plasminogen activator (t-PA). However any sign of deterioration must indicate more aggressive treatment.

The mainstay of treatment in all cases is to provide as rapidly as possible either relief of the obstruction or appropriate atlernative hepatic venous drainage. The former can be achieved by surgical removal or angioplastic dilatation of any caval or hepatic venous obstruction. The latter can be achieved by decompressing the portal hypertension. If this can be done before major hepatic damage occurs then the congestion is relieved, any liver damage is reversible, and the subsequent complications prevented. The paper by Dr Orloff and colleagues clearly supports this.

If decompression is not effected early then either hepatocellular failure and death or progression to chronic irreversible cardiac type cirrhosis will occur and such patients will need a liver transplant. However there is no substantiated evidence yet that liver transplantation is indicated in the absence of cirrhotic change[3].

There is much controversy regarding the method of providing adequate portal decompression. A side-to-side portal system to infra-hepatic vena cava shunt is effective providing there is significant pressure difference between the two systems. If the pressure in the two systems is equal which happens when there is total obstruction to the hepatic part of the inferior vena cava then such a shunt will be ineffective. To overcome this it is necessary to shunt the portal blood into a venous sytem with a lower pressure either above the obstruction or the right atrium. This requires a long prosthetic graft which carries a high risk of subsequent thrombosis.

To address this Dr Orloff has studied in animals and later clinically a combination of a side-to-side portacaval shunt with a cavo-atrial prosthetic shunt. He concludes this decompresses the liver and results in excellent flow in the long prosthetic graft with a low incidence of thrombosis due to high flow rates.

Another method to overcome the problem of high infra-hepatic caval pressure is to insert an expanding stent into the compressed or occluded part of the vessel[4]. This will restore caval blood flow, reduce the infrahepatic caval pressure and allow a subsequent side-to-side portacaval shunt to be constructed effectively without the need for a long prosthesis.

Unfortunately there are no controlled studies comparing the various decompression procedures either with each other or with liver transplantation. At the Third International Symposium on Budd-Chiari Syndrome to be held in London next year it is planned to discuss classification of the syndrome. Hopefully then rational treatment for each group of patients will be proposed and prospective studies of different treatment modalities will be possible.

Professor K.E.F. Hobbs
University Department of Surgery
The Royal Free Hospital School of Medicine
Pond Street
London NW3 2QG
United Kingdom

### REFERENCES

1. Hobbs, K.E.F. (1992) Conference — Budd-Chiari syndrome. *Lancet*, **339**, 115–116
2. Hamilton, G. and Dawson, K.J. (1992) Thrombolytic therapy for acute vascular emergencies. In: *Emergency Vascular Surgery*, (Eds) Greenhalgh, R.M. & Hollier, L. London: W.B. Saunders, pp. 37–54
3. Bismuth, H. and Sherlock, D.J. (1990) Portasystemic shunting versus liver transplantation for Budd-Chiari syndrome. *Annals of Surgery*, **214**, 581–589
4. Gillams, A., Dick, R., Platts, A., Irving, D. and Hobbs, K. (1991) Dilatation of the inferior vena cava using an expandable metal stent in Budd-Chiari syndrome. *Journal of Hepatology*, **13**, 149–151

